# Durum Wheat in Conventional and Organic Farming: Yield Amount and Pasta Quality in Southern Italy

**DOI:** 10.1100/2012/973058

**Published:** 2012-06-04

**Authors:** Massimo Fagnano, Nunzio Fiorentino, Maria Grazia D'Egidio, Fabrizio Quaranta, Alberto Ritieni, Rosalia Ferracane, Giampaolo Raimondi

**Affiliations:** ^1^Department of Agricultural Engineering and Agronomy, Naples University Federico II, Via Università 100, 80055 Portici, Naples, Italy; ^2^Research Unit for Cereal Quality (QCE), Agricultural Research Council (CRA), Via Cassia 176, 00191 Rome, Italy; ^3^Department of Pharmaceutical and Toxicological Chemistry, Naples University Federico II, Via D. Montesano 49, 80131 Naples, Italy; ^4^Department of Food Science, Naples University Federico II, Via Università 100, 80055 Portici, Naples, Italy; ^5^Torre Lama farm, Naples University Federico II, Via Fortunato, 84092 Bellizzi, Salerno, Italy

## Abstract

Five durum wheat cultivars were grown in a Mediterranean area (Southern Italy) under conventional and organic farming with the aim to evaluate agronomic, technological, sensory, and sanitary quality of grains and pasta. The cultivar Matt produced the best pasta quality under conventional cropping system, while the quality parameters evaluated were unsatisfactory under organic farming. The cultivar Saragolla showed the best yield amount and pasta quality in all the experimental conditions, thus proving to be the cultivar more adapt to organic farming. In all the tested experimental conditions, nivalenol (NIV) and deoxynivalenol (DON) occurrence was very low and the other mycotoxins evaluated were completely absent. These data confirm the low risk of mycotoxin contamination in the Mediterranean climate conditions. Finally, it has been possible to produce high-quality pasta in Southern Italy from durum wheat grown both in conventional and organic farming.

## 1. Introduction

Durum wheat (*Triticum durum* Desf.) is the most widespread crop in the Mediterranean area (1.636 million ha of cultivated area and 4.313 million tons of grain production). In Italy, about 67% of durum wheat production comes from the Southern regions and it is mainly used for pasta production [1].

The global quality of durum wheat belongs to the complex interaction between environmental conditions, yield characteristics, and technological requirements for its transformation in pasta. A worldwide agreement considers protein content and gluten quality the main factors that influence pasta quality [2, 3], but their relative weight depends on many factors, such as genotype, environment, and pasta processing conditions, like drying temperature [4, 5].

The quality of durum wheat may be evaluated by more than one point of view: agronomical quality that influences potentiality and stability of grain yield; milling quality that influences semolina yield, ash content, humidity, and impurity of grains; technological quality that influences content of protein and gluten quantity and quality; hygienic and sanitary quality that are related to some phytopathological microorganisms or their secondary metabolites. Recently, the consumer is also oriented toward other meanings of quality based on environmental and ethic friendly production.

The environmental impact of pasta production has been mainly related to the cropping system, the production techniques of semolina, and the adopted packaging [6]. In this perspective, the promotion of foods produced within short distance from the consumer by a local supply chain, the “zero-km,” may increase both environmental and economical sustainability. Organic farming represents another viable option for reducing environmental impact of pasta production [6].

Water deficit and high temperature are the main environmental factors affecting durum wheat grain quality under Mediterranean climate. It was reported that periods of heat stress with temperatures higher than 35°C during grain filling may alter flour, dough, and baking quality [7].

Contrasting results are reported about the relationships between water deficit and wheat quality. A spring water deficit could decrease N uptake and translocation to grains, thus reducing protein accumulation and grain quality [8]. On the other hand, water deficit during grain filling may increase aggregation level of glutenin subunits, improving grain technological quality [9].

Another very important aspect for both wheat and pasta quality is grain safety, with particular regards to mycotoxins, secondary metabolites produced by many genera of fungi. Mycotoxin presence depends on several factors, such as fungal strain, climatic and geographical conditions, and cultivation techniques [10].

Zearalenone and trichothecenes are produced by *Fusarium* species before or immediately after the harvest time. *Penicillium* and *Aspergillus *species generally grow during grain drying and subsequent steps and their risk is related to the production and accumulation of aflatoxins and ochratoxin A in wheat, maize, oat, barley, and other crops [11].

Mycotoxins present in durum wheat grains and derived products are mainly due to infections by *Fusarium graminearum *and *F. culmorum* [12].

In this work, the influence of cultivars and cropping system on agronomic, milling, technological, sensory, and sanitary quality of durum wheat and pasta has been evaluated within a local farm-to-table food chain.

## 2. Materials and Methods

### 2.1. Description of Site, Plant Cultivation, and Experimental Design

The experiment was carried out from November 2009 to July 2010 in the Torre Lama experimental farm of the Naples University Federico II, located in an alluvial plain of Southern Italy (40°37′N, 14°58′E, 30 m a.s.l.). The soil was subalkaline (pH 7.5), silty-clay-loam (Clay 334 g kg^−1^, Silt 241 g kg^−1^, Sand 425 g kg^−1^) with low N and SOM content (1.2 g kg^−1^ and 18.4 g kg^−1^, resp.).

Five durum wheat cultivars (Matt, Karalis, Pablo, San Carlo, and Saragolla) were grown under conventional (mineral fertilization and chemical weed control) and organic (organic fertilization and no weed control) cropping systems in a farm scale experiment, arranging 1 ha plots in a complete randomized scheme with 2 replications. Therefore, the experiment was carried out on a 20 ha total area (5 cultivars × 2 cropping systems × 2 replications).

The preceding crop was corn in all the plots. Soil tillage was made on August 25, sowing on November 5, top dressing and chemical weed control (only on conventional plots) on March 22, and then harvest on June 29. The long time between soil tillage and sowing is usual in Mediterranean area where the main tillage (moldboard plowing) is done when the soil is well dry in all the 30–40 cm tillage layer (i.e., at the end of summer), while the seed-bed preparation (rotary hoeing) and sowing is made following the first autumn rainfalls (i.e., at November) when soil moisture become adequate for seed germination.

The N supply was calculated taking into account both uptake (27.2 kg N Mg^−1^ grain yield) and expected grain yield (4 Mg ha^−1^) suggested by the Regional Department of Agriculture. In order to improve grain quality and to reduce nitrate pollution, the soluble fertilizers were supplied after the rainy period, at the end of tillering.

In conventional cropping system, weed control was made with Atlantis WG (mefenpir-Dietile 90 g kg^−1^, Iodosulfuron-Metile-Sodio 6 g kg^−1^, Mesosulfuron-Metile 30 g kg^−1^, Bayer) mixed with Granstar 50 SX (Tribenuron metile 500 g kg^−1^, DuPont); fertilization was made with 88 kg ha^−1^ of Diammonium Phosphate (18.46.0) at sowing and 202 kg ha^−1^ of Urea (46.0.0) at the end of tillering (March 22).

In organic cropping system, chemical weed control was not made and fertilization was made at seeding with 1173 kg ha^−1^ of Naturale (8.8.6., Fertenia, Bellizzi, Italy), a fertilizer made with vegetable, meat and feather meal, and with 375 kg ha^−1^ of Borlanda (3.0.6, Fertenia, Bellizzi, Italy), a liquid fertilizer made from grape-vinasse, at the end of tillering (March 22).

**Figure 1:**
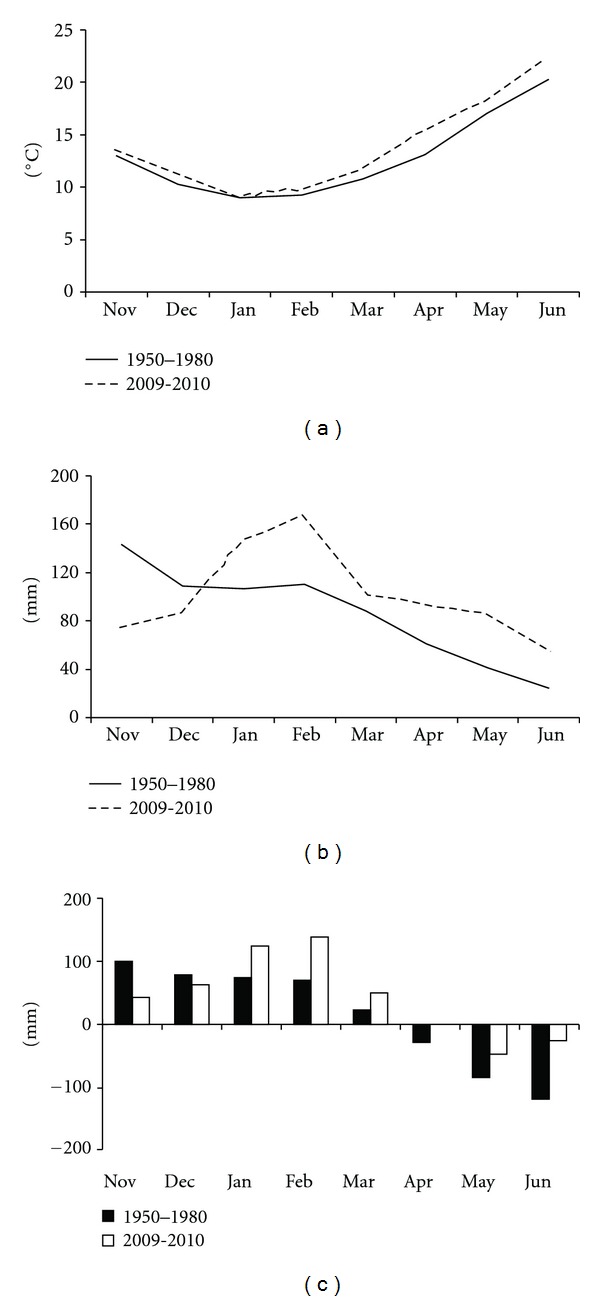
Average temperature (a), rainfalls (b), and water balance (c) during the growth period of wheat: 2009-2010 versus 1950–1980.

During 2009-2010 (Figure 1(a)), temperatures were higher than the long term (1950–1980) average values (+1.0°C on the average). The highest value (35°C) was recorded at the half of June, while values below zero were recorded on December 21, January 24, and February 2. Rainfalls (Figure 1(b)) were higher than the long term values (+37 mm from November to June), particularly at the end of winter (+40 mm on January and +58 on February). Water balance was calculated as the difference between rainfall and ETmax which, in turn, was calculated as the product of the daily reference evapotranspiration [13] and the crop coefficients [14]. According with the water balance analysis (Figure 1(c)), the year of this experiment (2009-2010) was more humid than the long-term average (1950–1980): water surplus from sowing (November) to stem extension (April) was 417 mm (2009-2010) versus 313 mm (1950–1980). Water deficit during 2009-2010 spring period was more moderate than the long-term average: during heading (May) the values were −48 mm and −86 mm, respectively, and during ripening (June) were −26 mm and −119 mm, respectively.

### 2.2. Agronomic Measurements

Measurements of yield components of wheat (total biomass weight, plant height, grain weight, harvest index, number of spikes per m^2^, number of grains per spike, grain average weight, % yellowberry, test weight) were made at harvest, on 3 sample areas (10 m^2^) per plot.

### 2.3. Grain and Pasta Quality Measurements

Quality analyses were carried out on grains, wholemeal, semolina, and pasta.

Test weight was determined on the grain samples by automatic instrument Infratec Grain Analyzer 1241 (FOSS AB Analytical, Sweden). The grains were milled to wholemeal with a Cyclotec mill-PBI (Italy) and sieved with a 1 mm mesh; the protein contents were determined by Dumas combustion method (ICC method n. 167) with automatic instrument Leco FP 428 (USA). Sedimentation index was evaluated in sodium-dodecyl-sulphate, according with the ICC method n. 151 with SDS 3%.

Semolina samples, obtained by a pilot milling plant (Buhler MLU 202) with three breaking and three sizing passages, were subjected to the following quality analyses: protein content, gluten quality, and color. Protein content was determined by Dumas combustion method, gluten content was determined according to EN ISO 21415 method. Gluten quality was evaluated by Gluten Index direct method by using Glutomatic System (Perten, Sweden) (ICC 158) and by an indirect method using the alveograph Chopin (UNI 10453 method). Semolina color, expressed by yellow and brown index, was evaluated by reflection colorimeter Minolta Chromameter CR-400 (illuminating D65) combined with CR-A50 for the measurements of granular material.

Semolina was also used to produce pasta samples (spaghetti shape, 1.65 mm diameter) by an experimental press (Namad, Italy) and by an experimental drying system (AFREM-France) employing a low-temperature drying diagram (50°C). Pasta cooking quality was assessed by sensory analysis both at the optimal cooking time (OCT) corresponding to the disappearance of starchy central core of spaghetti, and at a standard cooking time of 13 min (SCT) which corresponds to an overcooking for 1.65 mm diameter spaghetti. The sensorial judgment (SJ) is based on three textural characteristics: firmness, stickiness, and bulkiness [15]. Stickiness is the material adhering to the surface of cooked pasta; bulkiness is the adhesion degree of pasta stands to each other; firmness represents the resistance of cooked pasta to chewing by teeth. Each of these three parameters was evaluated by a score ranging from 10 to 100. For stickiness and bulkiness, ≤ 20 = very high, 40 = high, 60 = rare, 80 = almost absent, 100 = absent; for firmness, ≤ 20 = absent, 40 = rare, 60 = sufficient, 80 = good, 100 = very good. The score of each sensorial component was the arithmetic mean of three assessors; the final value of SJ was the arithmetic mean of the three textural components: the higher is the SJ value, the greater higher is the quality.

### 2.4. Mycotoxins Analyses

Trichothecenes standards were purchased from Sigma-Aldrich (Milan, Italy) and stored at 4°C in the dark. Each standard was dissolved in methanol at 1 mg/mL concentration. Working solution and successive dilutions were prepared with solvent mixture (CH_3_OH/H_2_O, 70 : 30, v/v).

Water for LC mobile phase and organic solvents were HPLC grade from Merck (Darmstadt, Germany), ammonium acetate (MS grade) was purchased from Sigma-Aldrich, formic acid (p.a.) was obtained from Fluka (Milan, Italy). The extracts were filtered with Whatman no.4 type filter papers (Maidstone, England). Filtek syringe filters (0.22 *μ*m; 25 mm) were provided from Chemtek Analytica (Bologna, Italy).

Sample preparation and analysis were performed according to Santini and others [16]. The simultaneous extraction of mycotoxins was performed by adding 50 mL of a CH_3_CN/H_2_O (84 : 16; v/v) mixture to a vessel containing 10 g of finely grounded sample and stirred for 1 h. The obtained extract phase was paper filtered and 5 mL of the filtrate were recovered and evaporated using a centrifugal evaporator (Savant). The dry extracts were then dissolved in 1 mL of CH_3_OH/H_2_O mixture (70 : 30, v/v) and finally filtered through a 0.22 *μ*m cellulose filter (Chemtek Analytica, Bologna, Italy) before LC-MS-MS analysis.

LC analysis was performed using a system consisting of two micropumps (Series 200, PerkinElmer, Waltham, MA, USA). A Gemini column (3 *μ*m C18 110A, 150 × 4.60 mm, Phenomenex, USA) heated to 50°C was used; the flow rate was set to 0.8 mL/min and the injection volume was 20 *μ*L.

Mobile phase A consisted of an H_2_O/CH_3_OH mixture (90 : 10, v/v) containing 5 mmol L^−1^ ammonium acetate, while mobile phase B consisted of a CH_3_OH/H_2_O mixture, also containing 5 mmol L^−1^ ammonium acetate (90 : 10, v/v). The following binary gradient was applied: initial condition 10% B; 0–7 min, 35% B; 7–9 min, 80% B; 9–13 min constant at 80% B; 13–15 min 100% B, finally returning to the initial conditions in 3 min.

MS/MS data were obtained using an API 3000 triple-quadrupole mass spectrometer (Applied Biosystems, Ont, Canada) equipped with an APCI interface, using the following settings: probe temperature 450°C, corona current (NC) ±2 *μ*A (depending on use in positive or negative mode).

The declustering potential (DP) and collision energy (CE) were optimized for each compound by direct infusion of standard solutions (10 *μ*g/mL) into the mass spectrometer at a flow rate of 8 *μ*L/min, using a Model 11 syringe pump (Harvard Apparatus, Holliston, MA, USA).

The acquisition was performed in Multiple Reaction Monitoring (MRM) both in the negative and positive ion, depending on chemical structure of each compound.

In Table 1, LC/MS/MS characteristics of each analyzed compound are reported.

### 2.5. Statistical Analyses

A two factor completely randomized design with two replications was used to assess the effects of cultivars and cropping systems. Analysis of variance (ANOVA) was performed by the MSTAT-C software (Crop and Soil Science Department, Michigan State University, Version 2.0). Means of the factors that resulted significant from ANOVA (*F*-test) were separated by calculating LSD per *P* < 0.05.

## 3. Results and Discussion

### 3.1. Agronomic Results

The interaction between cropping systems and cultivars was not significant for grain yield (Table 2) and yield components (Table 3), therefore, only the average values of main factors (cultivars and cropping systems) will be presented and discussed.

Wheat yield (Table 2) with organic cropping system was 21% lower than with conventional (2.5 versus 3.2 Mg ha^−1^ on the average) confirming the results reported in other field experiments [17].

The highest yield was obtained by Saragolla (3.8 Mg ha^−1^), while the values were significantly lower for the other cultivars (2.5–2.9 Mg ha^−1^).

Though the interaction cropping system by cultivar was not significant according with ANOVA, a different behavior of the cultivar is identifiable with lower yield losses under the organic system, as compared to the conventional one, for Saragolla and Pablo (−11 and −14% resp.). This suggests that those two cultivars may be more suitable to organic farming and confirms the importance of genotype selection for adaptability to organic farming [18].

Total biomass yield (Table 3) was higher in Karalis followed by Pablo and Saragolla, while the effect of cropping system was not significant. Harvest index was higher under the conventional system and, among the cultivars, Saragolla and San Carlo gave higher values.

The number of spikes per m^2^ was very high on the average, particularly for the cultivars Matt, Pablo, and San Carlo, while the average weight of spikes was very low mainly for Matt and Pablo. The average weight of kernels was low on the average, with the exception of Karalis. The effect of cropping system on these parameters was not significant.

The percentage of yellowberry was not different among the cultivars, while it was significantly higher under organic conditions systems (35.6% versus 10.6% on the average).

The hectoliter weight was higher in Karalis and Matt and lower in Pablo, while it was not related to the kind of fertilization. In any case it was higher than 80 that is considered the minimum threshold by the major pasta factories.

### 3.2. Grain and Pasta Quality

The results of quality assessment (Table 4) pointed out that the cultivars Matt and Karalis, under conventional cropping system, reached the highest values for protein, gluten content, gluten quality, and alveographic W values. The gluten index was high (>75) for all the cultivars, thus showing a lower differentiating power.

The results obtained from pasta cooking evaluation (Table 5) showed that Saragolla under conventional cropping system gained the highest quality level in OCT conditions, followed by Matt and Karalis; Saragolla had the best performance in organic farming too, together with San Carlo. The sensorial judgment in SCT conditions showed a different behavior of the cultivars: under conventional system, Matt reached the highest values, while in organic farming, Saragolla maintained the top position. Matt and San Carlo in conventional and Pablo and Saragolla in organic were the cultivars with lower differences between the two different cooking times, thus showing a higher resistance to overcooking (Table 6).

The comparison between the two cropping systems showed that the organic system determined a reduction of protein content, gluten content, and quality as evidenced by protein level and SDS test in wholemeal and by protein content, gluten content, and alveographic W values in semolina, thus confirming the well-known effects of N nutrition on the quality parameters [19]. These results confirm that the main problem for wheat from organic farming is the low N availability during reproductive phases that reduces protein accumulation in grains [20], as it has been found in many other foods [21]. Only the management of rotations including legume crops may improve soil N content, at sustainable economical costs, thus reducing the N nutrition deficiency under organic farming conditions [22, 23].

Pasta cooking quality with organic system reached lower scores in comparison with conventional system. In OCT experimental conditions, the reduction of pasta quality from organic wheat was similar among the cultivars (from −16 to −18), except for San Carlo that showed a little increase in the pasta quality score (+5). On the contrary, when SCT was used (i.e., in overcooking conditions), the quality decrease was very high for Karalis and Matt (−15 and −29 resp.), while Pablo, San Carlo, and Saragolla were somewhat unaffected by the cropping systems. These differences were already expected considering the different varietal responses to the lower levels of protein content and gluten quality, that are widely recognized as the most important parameters for pasta processing, mainly when low temperature drying cycles were used [4].

Comparison between productive and qualitative parameters showed a positive correlation between grain yield and pasta quality (*r* = 0.68; *P* ≤ 0.05), thus indicating that an adequate N supply can gain the best results both for quantitative and qualitative aspects.

As expected [24], yellowberry was negatively correlated with protein content (*r* = −0.73; *P* ≤ 0.05). Pasta quality was positively correlated with protein (*r* = 0.84; *P* ≤ 0.01), gluten content (*r* = 0.65; *P* ≤ 0.05), and alveographic W (*r* = 0.66; *P* ≤ 0.05): such correlations confirm that the above mentioned parameters are the most important ones for high-quality pasta production.

### 3.3. Mycotoxin Contamination

The average level of Nivalenol was about 20 *μ*g kg^−1^ without substantial and statistical differences between conventional and organic cropping systems (15.0 *μ*g kg^−1^and 25.3 *μ*g kg^−1^, resp.), according to the results of Quaranta and others [25]. Among the varieties, the higher values were measured in Pablo and San Carlo (48.2 and 23.7 *μ*g kg^−1^ resp.), and the lower in Saragolla, Matt, and Karalis (13.0, 9.9, and 6.3 *μ*g kg^−1^ resp.). The contamination level was clearly below the Tolerable Daily Intake (TDI) for humans (700 mg kg^−1^) [26].

On the contrary high Nivalenol contamination in wheat grain occurred in more rainy areas: Ioos and others [27] found a level of 100–595 *μ*g kg^−1^ in durum wheat samples collected in different regions of France.

Deoxynivalenol concentration averaged 70.8 *μ*g kg^−1^ in conventional and 26.7 *μ*g kg^−1^ in organic samples, without significant differences among the varieties.

A study conducted in Italy, showed a decreasing deoxynivalenol contamination from Northern to Southern regions [28]. These results were confirmed by Gallo and others [29] who found in Southern Italy (Sicily) an average contamination of 300 *μ*g kg^−1^ and 100 *μ*g kg^−1^, respectively, in 2005 and 2006 crop seasons.

On the contrary, in Northern Italy the deoxynivalenol content during the rainier year was higher than the admissible maximum levels (1750 mg kg^−1^) based on UE Commission Regulation (EC) 1881/2006 of December 19, 2006 [30].

Fusarenon X, neosalaniol,T-2 toxin, HT-2 toxin, diacetoxyscirpenol, and 3-acetyldeoxynivalenol in analyzed samples were below the detection limit.

Literature data report that the main producers of T-2, HT-2 and neosolaniol (*Fusarium sporotrichioides* and *Fusarium poae*) are widespread in cold European areas. In Poland, T-2 and H-T2 occurrence is reported in wheat (up to 2400 and 370 *μ*g kg^−1^ resp.) and in Germany, levels of T2 of 5 to 600 *μ*g kg^−1^were found in 38% of wheat samples [28].

The data about mycotoxins contamination obtained in this experiment clearly show that durum wheat cultivated in the climatic conditions of Southern Italy is safer than in Northern Italy or Central Europe, confirming the results of other studies [31]. It is noteworthy that these results were obtained in a year more humid than the average.

## 4. Conclusions

The production of durum wheat for high-quality pasta in Southern Italy was mainly affected by the different responses of cultivars to the lower N availability caused by organic cropping system.

Higher N availability during the spring, belonging to mineral fertilization at the end of tillering (March), allowed Matt to produce the best quality pasta, while its performance was unsatisfactory under organic farming conditions.

Saragolla showed the highest yield and a high pasta quality in all the experimental conditions, thus proving to be a cultivar very adaptable to the lower N availability occurred under organic farming.

In conventional cropping systems, fractional application of N fertilizer is mandatory to gain the best results in terms of pasta quality, concentrating the highest dose at the end of rainy season (March, in Mediterranean environments). The scheme used in this experiment (25% of the total N uptake at sowing and 75% at the end of tillering) resulted appropriate to this aim.

In particularly rainy years, an increase of the N spring dose may be necessary. On the contrary, in drier seasons, irrigation (about 30 mm) at the end of stem elongation-boot stage could help the N uptake by plant. In this experiment, this was not necessary, since the rainfalls in May-June (141 mm) were able to allow a regular N uptake.

In organic cropping systems, the supply of a soluble fertilizer (vinasse) at the end of tillering was not sufficient to allow the adequate N availability for all the cultivars. In this case only, the choice of an adaptable genotype, such as Saragolla, can allow gaining satisfactory results in terms of yield amount and pasta quality. Nevertheless, only the presence of legume crops in long-term crop rotations could guarantee the necessary N availability at sustainable costs.

In these experimental conditions, very low levels of nivalenol (NIV) and deoxynivalenol (DON) were measured in wheat grains, with values much lower than the thresholds of European legislation (1750 *μ*g kg^−1^: EC Reg 1126/2007), completely absent resulted fusarenon X (FUS-X), Neosalanione (NEOS), diacetoxyscirpenol (DAS), T2-Toxin, HT2-Toxin, and acetyldesoxynivalenol (AcDON).

These results confirmed the importance of the environmental conditions on mycotoxin contamination of durum wheat, that is, a problem only in more humid areas (such as Northern Italy and Central Europe), while in Southern Italy, durum wheat grains are generally free from this kind of contamination, also in a year that was more rainy than the long-term average values, such as that of this experiment. The production from organic cropping system was also mycotoxin free.

All the data collected in this experiment show that the environmental conditions of Southern Italy allow to produce durum wheat and pasta of high quality both in terms of safety and organoleptic characteristics.

These results support the creation of a short food chain (so called “km zero”) that minimizes the environmental impact of all the production phases by reducing emissions related to the transport.

## Figures and Tables

**Table 1:** LC/MS/MS characteristics of the analyzed compounds.

Analyte	Retention time (min)	Precursor ion	Product ions	DP	CE
NIV	6.4	371.1 [M + CH_3_COO]^−^	59 281	35	15 22
DON	8.9	355.1 [M + CH_3_COO]^−^	59 265	31	45 19
3-AcDON	12.05	397.2 [M + CH_3_COO]^−^	59 307	31	42 21
Fus X	11.2	413.3 [M + CH_3_COO]^−^	59 187	28	42 38
NEO	11.4	400.1 [M + NH_4_]^+^	305.1 244.9	40	17 17
HT-2	14.6	442.4 [M + NH_4_]^+^	263 215	30	18 18
T-2	15.9	484.2 [M + NH_4_]^+^	305.2 185	35	20 29
DAS	13.4	384.2 [M + NH_4_]^+^	307.2 105	40	17 49

**Table 2:** Grain yield: effect of cropping system and cultivar.

Cropping system cultivar	Organic	Conventional	Average	Difference in organic
	Mg ha^−1^	Mg ha^−1^	Mg ha^−1^	%
Matt	2.13	2.85	2.48 b	−25.0
Saragolla	3.62	4.09	3.85 a	−11.4
Karalis	2.33	3.22	2.78 b	−27.6
Pablo	2.57	2.99	2.78 b	−14.2
San Carlo	2.46	3.45	2.94 b	−28.8

Average	2.52 b	3.23 a	2.87	−21.0

Different letters indicate significant differences per *P* ≤ 0.05.

**Table 3:** Yield components: effect of cropping system and cultivar.

	Biomass Yield	Plant Height	Harvest Index	Spikes		Average weight	Yellowberry	Hectoliter weight
	Mg ha^−1^	cm	%	Num·m^−2^	g spike^−1^	mg grain^−1^	%	g Hl^−1^
Cultivar								
Matt	10.8 b	63 a	24.0 b	573 a	0.76 c	43.1 b	21.5	84 a
Saragolla	11.9 ab	67 a	32.1 a	334 c	1.41 a	48.5 b	14.8	82 b
Karalis	14.2 a	52 b	20.6 b	341 c	1.10 b	57.6 a	22.9	85 a
Pablo	12.6 ab	51 b	22.5 b	491 ab	0.58 c	48.4 b	26.0	79 c
San Carlo	10.1 b	56 ab	29.1 a	448 abc	1.00 b	49.5 b	30.5	81 bc

*Significance*	*0.05*	*0.01*	*0.01*	*0.02*	*0.01*	*0.01*	*n.s.*	*0.01*

Cropping system								
Organic	12.3	61.5 a	23.5 b	415	0.96	48.8	35.6 a	82
Conventional	11.6	54.4 b	27.9 a	461	0.98	50.2	10.6 b	83

*Significance*	*n.s.*	*0.02*	*0.02*	*n.s.*	*n.s.*	*n.s.*	*0.01*	*n.s.*

Different letters indicate significant differences per *P* ≤ 0.05, n.s.: not significant.

**Table 4:** Quality parameters of wholemeal and semolina of durum wheat varieties cultivated with conventional and organic cropping systems.

Cultivar	Wholemeal		Semolina						
	Protein g kg^−1^ d.m.	SDS ml	Protein g kg^−1^ d.m.	Gluten g kg^−1^ d.m.	Gluten Index	W	P/L	Yellow index	Brown index
Conventional cropping system									
Karalis	146	57	133	111	84	217	0.85	18.8	10.0
Matt	163	64	140	118	75	232	0.81	26.3	10.5
Pablo	122	60	115	96	88	216	0.81	28.5	10.6
San Carlo	119	50	103	84	95	180	2.10	24.0	10.1
Saragolla	133	49	126	93	86	191	1.37	25.2	12.3

*Average *	*137*	*56*	*123*	*100*	*86*	*207*	*1.19*	*24.6*	*10.7*
*S.D.*	*18*	*6*	*15*	*14*	*7*	*21*	*0.6*	*3.6*	*0.9*

Organic cropping system									
Karalis	101	42	95	75	95	145	1.23	17.0	8.1
Matt	104	41	97	77	89	156	1.18	22.3	8.3
Pablo	115	46	105	90	83	183	1.13	27.2	10.5
San Carlo	115	51	103	85	93	178	2.20	22.8	10.1
Saragolla	120	42	108	81	94	159	1.71	23.5	10.2

*Average *	*111*	*44*	*102*	*81*	*91*	*164*	*1.49*	*22.6*	*9.4*
*S.D.*	*8*	*4*	*5*	*6*	*4*	*16*	*0.5*	*3.7*	*1.1*

Variation (%) with organic cropping system									
Karalis	−30.3	−26.3	−28.7	−32.7	13.1	−33.2	44.7	−9.6	−18.3
Matt	−36.5	−35.9	−30.5	−34.8	18.7	−32.8	45.7	−15.2	−21.0
Pablo	−6.0	−23.3	−8.6	−7.2	−5.7	−15.3	39.5	−4.7	−1.1
San Carlo	−3.5	2.0	0.5	0.6	−2.1	−1.1	4.8	−5.0	0.5
Saragolla	−10.3	−14.3	−14.8	−13.2	9.3	−16.8	24.8	−6.6	−17.2

*Average*	−*18.9 *	−*20.7 *	−*17.7 *	−*19.1 *	*6.1*	−*20.8 *	*25.4*	−*8.2 *	−*11.6 *

S.D.: Standard deviation.

**Table 5:** Cooking quality parameters of pasta from durum wheat varieties cultivated with conventional and organic cropping systems.

Cultivar	Optimal cooking time (OCT)					Standard Cooking time (SCT = 13 min)			
	Time	Firmness	Stickiness	Bulkiness	Final judgment	Firmness	Stickiness	Bulkiness	Total judgment
Conventional cropping system									
Karalis	9′20′′	57	80	57	**65**	20	73	43	**45**
Matt	9′45′′	57	85	60	**67**	50	72	55	**59**
Pablo	10′15′′	55	78	55	**63**	25	68	45	**46**
San Carlo	9′30′′	45	75	52	**57**	38	68	47	**51**
Saragolla	9′05′′	72	80	65	**72**	42	63	50	**52**

*Average *	*9*′*36*′′	*57*	*80*	*58*	***65***	*35*	*69*	*48*	***51***
*S.D.*	*27*′′	*10*	*4*	*5*	***6***	*12*	*4*	*5*	***5***

Organic cropping system									
Karalis	9′40′′	42	72	45	**53**	10	57	35	**34**
Matt	9′20′′	48	70	50	**56**	18	65	43	**42**
Pablo	9′35′′	40	73	45	**53**	32	65	45	**47**
San Carlo	9′50′′	55	73	53	**60**	23	72	45	**48**
Saragolla	9′45′′	50	75	57	**61**	33	73	45	**53**

*Average *	*9*′*36*′′	*47*	*73*	*50*	***57***	*23*	*66*	*43*	***45***
*S.D.*	*12*′′	*6*	*2*	*5*	***4***	*10*	*6*	*4*	***7***

Variation (%) with organic cropping system									
Karalis	4	−26	−10	−21	**−18**	−50	−22	−19	**−25**
Matt	−4	−16	−18	−17	**−17**	−64	−10	−22	**−29**
Pablo	−7	−27	−6	−18	**−16**	28	−4	0	**3**
San Carlo	4	22	−3	2	**5**	−39	6	6	**−5**
Saragolla	7	−31	−6	−12	**−16**	−21	16	6	**3**

*Average*	1	−18	−9	−13	**−13**	−34	−3	−6	**−11**
*S.D.*	6	22	6	9	**10**	36	15	14	**15**

S.D.: Standard deviation.

**Table 6:** Resistance to overcooking expressed as the difference between SCT and OCT.

Cultivar	Firmness	Stickiness	Bulkiness	Total judgment
Conventional cropping system				
Karalis	−37	−7	−14	**−20**
Matt	−7	−13	−5	**−8**
Pablo	−30	−10	−10	**−17**
San Carlo	−7	−7	−5	**−6**
Saragolla	−30	−17	−15	**−20**

*Average *	−*22 *	−*11 *	−*10 *	**−*14 ***
*S.D.*	*14*	*4*	*5*	***7***

Organic cropping system				
Karalis	−32	−15	−10	**−19**
Matt	−30	−5	−7	**−14**
Pablo	−8	−8	0	**−6**
San Carlo	−32	−1	−8	**−12**
Saragolla	−17	−2	−12	**−8**

*Average *	−*24 *	−*6 *	−*7 *	−***12***
*S.D.*	*11*	*6*	*5*	***5***

S.D.: Standard deviation.

## References

[1] D’Egidio MG (2007). Overview on pasta in the world. *Tecnica Molitoria International*.

[2] Cubadda RE, Carcea M, Marconi E, Trivisonno MC (2007). Influence of gluten proteins and drying temperature on the cooking quality of durum wheat pasta. *Cereal Chemistry*.

[3] Sissons MJ, Egan NE, Gianibelli MC (2005). New insights into the role of gluten on durum pasta quality using reconstitution method. *Cereal Chemistry*.

[4] D’Egidio MG, Mariani BM, Nardi S, Novaro P (1990). Chemical and technological variables and their relationships: a predictive equation for pasta cooking quality. *Cereal Chemistry*.

[5] Novaro P, D’Egidio MG, Mariani BM, Nardi S (1993). Combined effect of protein content and high-temperature drying systems on pasta cooking quality. *Cereal Chemistry*.

[6] Bevilacqua M, Braglia M, Carmignani G, Zammori FA (2007). Life cycle assessment of pasta production in Italy. *Journal of Food Quality*.

[7] Blumenthal CS, Barlow EWR, Wrigley CW (1993). Growth environment and wheat quality: the effect of heat stress on dough properties and gluten proteins. *Journal of Cereal Science*.

[8] Garrido-Lestache E, López-Bellido RJ, López-Bellido L (2004). Effect of N rate, timing and splitting and N type on bread-making quality in hard red spring wheat under rainfed Mediterranean conditions. *Field Crops Research*.

[9] Flagella Z, Giuliani MM, Giuzio L, Volpi C, Masci S (2010). Influence of water deficit on durum wheat storage protein composition and technological quality. *European Journal of Agronomy*.

[10] Lo Curto R, Pellicano T, Vilasia F, Munafo P, Dugo G (2004). Ochratoxin A occurrence in experimental wines in relationship with different pesticide treatments on grapes. *Food Chemistry*.

[11] Aresta A, Cioffi N, Palmisano F, Zambonin CG (2003). Simultaneous determination of ochratoxin A and cyclopiazonic, mycophenolic, and tenuazonic acids in cornflakes by solid-phase microextraction coupled to high-performance liquid chromatography. *Journal of Agricultural and Food Chemistry*.

[12] Logrieco A, Bottalico A, Mulé G, Moretti A, Perrone G (2003). Epidemiology of toxigenic fungi and their associated mycotoxins for some Mediterranean crops. *European Journal of Plant Pathology*.

[13] Hargreaves GL, Hargreaves GH, Riley JP (1985). Agricultural benefits for Senegal river basin. *Journal of Irrigation & Drainage Engineering*.

[14] Allen RG, Pereira LS, Raes D, Smith M (1998). Crop evapotranspiration. guidelines for computing crop water requirements. *FAO Irrigation Drainage Paper*.

[15] D’Egidio MG, Mariani BM, Nardi S, Novaro P (1993). Viscoelastograph measures and total organic matter test: suitability in evaluating textural characteristics of cooked pasta. *Cereal Chemistry*.

[16] Santini A, Ferracane R, Somma MC, Aragónb A, Ritieni A (2009). Multitoxin extraction and detection of trichothecenes in cereals: an improved LC-MS/MS approach. *Journal of the Science of Food and Agriculture*.

[17] Mader P, Hahn D, Dubois D (2007). Wheat quality in organic and conventional farming: results of a 21 year field experiment. *Journal of the Science of Food and Agriculture*.

[18] Murphy KM, Campbell KG, Lyon SR, Jones SS (2007). Evidence of varietal adaptation to organic farming systems. *Field Crops Research*.

[19] Flagella Z (2006). Nutritional and technological quality of the durum wheat. *Italian Journal of Agronomy*.

[20] Woese K, Lange D, Boess C, Bogl KW (1997). A comparison of organically and conventionally grown foods: results of a review of the relevant literature. *Journal of the Science of Food and Agriculture*.

[21] Winter CK, Davis SF (2006). Organic foods. *Journal of Food Science*.

[22] Ryan MH, Derrick JW, Dann PR (2004). Grain mineral concentrations and yield of wheat grown under organic and conventional management. *Journal of the Science of Food and Agriculture*.

[23] Tosti G, Guiducci M (2010). Durum wheat-faba bean temporary intercropping: effects on nitrogen supply and wheat quality. *European Journal of Agronomy*.

[24] Gianibelli MC, Arango C, Sarandon SJ, Bushuk W, Tkachuk R (1990). Protein composition of vitreous and yellow berry bread wheat—influence of nitrogen-fertilization. *Gluten Proteins*.

[25] Quaranta F, Amoriello T, Aureli G (2010). Grain yield, quality and deoxinyvalenol (DON) contamination of durum wheat (*Triticum durum* Desf.): results of national networks in organic and conventional cropping systems. *Italian Journal of Agronomy*.

[26] EC *Opinion of the Scientific Committee on Food on Fusarium toxins. Part 6: Group evaluation of T-2 toxin, HT-2 toxin, nivalenol and deoxynivalenol*.

[27] Ioos R, Belhadj A, Menez M, Faure A (2005). The effects of fungicides on Fusarium spp. and Microdochium nivale and their associated trichothecene mycotoxins in French naturally-infected cereal grains. *Crop Protection*.

[28] Bottalico A, Perrone G (2002). Toxigenic Fusarium species and mycotoxins associated with head blight in small-grain cereals in Europe. *European Journal of Plant Pathology*.

[29] Gallo G, Lo Bianco M, Bognanni R, Saimbene G (2008). Mycotoxins in durum wheat grain: hygienic-health quality of Sicilian production. *Journal of Food Science*.

[30] Blandino M, Pilati A, Reyneri A (2009). Effect of foliar treatments to durum wheat on flag leaf senescence, grain yield, quality and deoxynivalenol contamination in North Italy. *Field Crops Research*.

[31] Bognanni R, Gallo G, Grillo O, Ribilotta MG (2005). Monitoring of mycotoxins in Sicilian durum wheat. *Tecnica Molitoria*.

